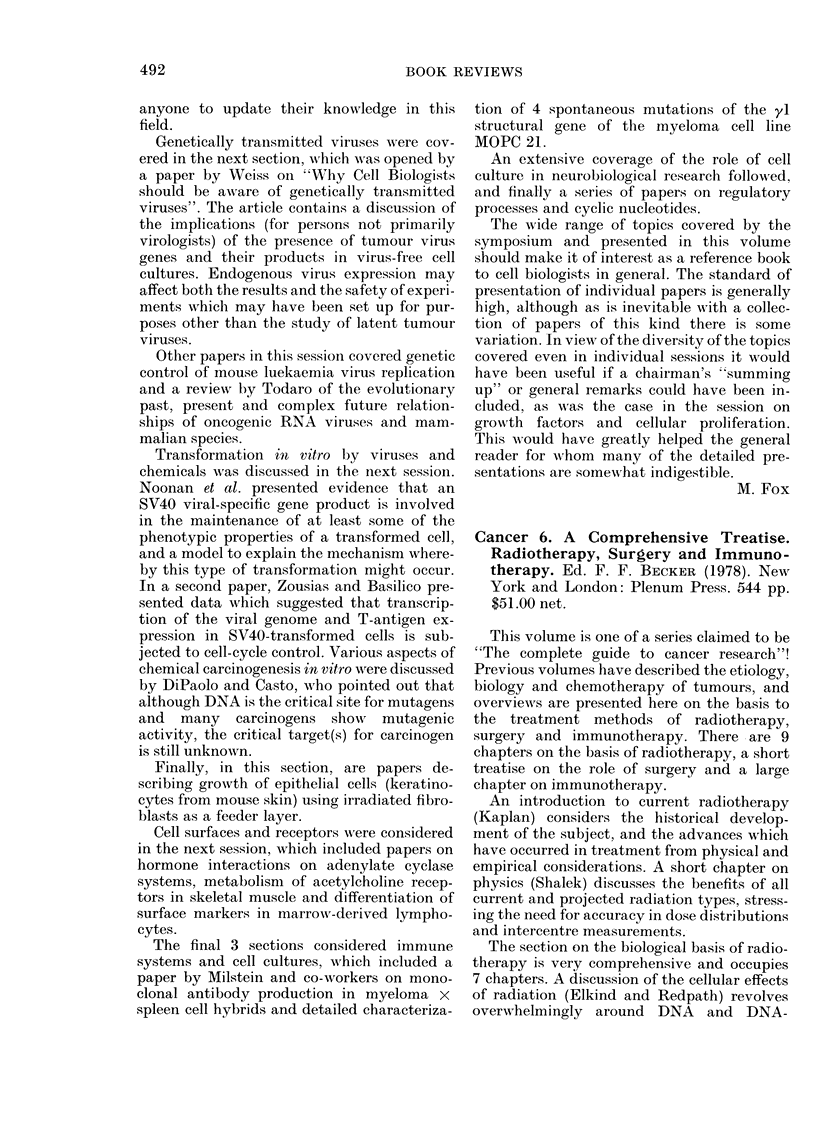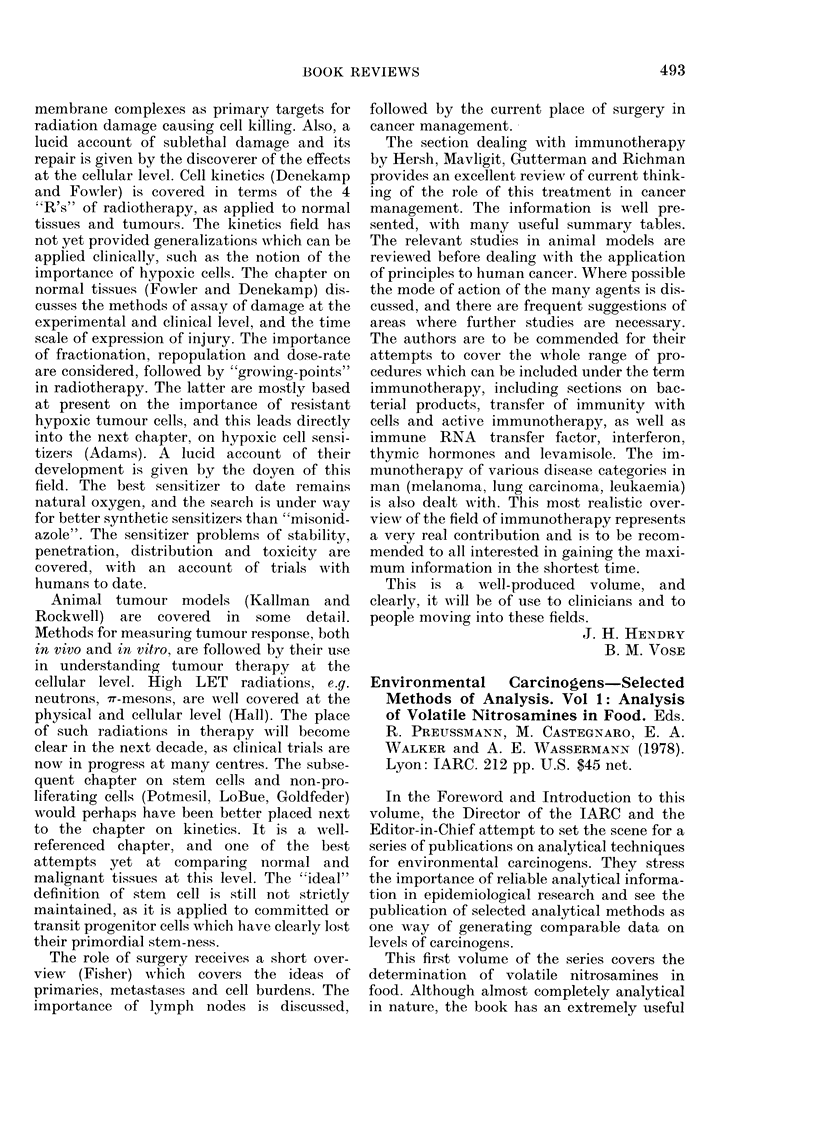# Cancer 6. A Comprehensive Treatise. Radiotherapy, Surgery and Immunotherapy

**Published:** 1979-04

**Authors:** J. H. Hendry, B. M. Vose


**Cancer 6. A Comprehensive Treatise. Radiotherapy, Surgery and Immunotherapy.** Ed. F. F. Becker (1978). New York and London: Plenum Press. 544 pp. $51.00 net.

This volume is one of a series claimed to be "The complete guide to cancer research"! Previous volumes have described the etiology, biology and chemotherapy of tumours, and overviews are presented here on the basis to the treatment methods of radiotherapy, surgery and immunotherapy. There are 9 chapters on the basis of radiotherapy, a short treatise on the role of surgery and a large chapter on immunotherapy.

An introduction to current radiotherapy (Kaplan) considers the historical development of the subject, and the advances which have occurred in treatment from physical and empirical considerations. A short chapter on physics (Shalek) discusses the benefits of all current and projected radiation types, stressing the need for accuracy in dose distributions and intercentre measurements.

The section on the biological basis of radiotherapy is very comprehensive and occupies 7 chapters. A discussion of the cellular effects of radiation (Elkind and Redpath) revolves overwhelmingly around DNA and DNA-

membrane complexes as primary targets for radiation damage causing cell killing. Also, a lucid account of sublethal damage and its repair is given by the discoverer of the effects at the cellular level. Cell kinetics (Denekamp and Fowler) is covered in terms of the 4 "R's" of radiotherapy, as applied to normal tissues and tumours. The kinetics field has not yet provided generalizations which can be applied clinically, such as the notion of the importance of hypoxic cells. The chapter on normal tissues (Fowler and Denekamp) discusses the methods of assay of damage at the experimental and clinical level, and the time scale of expression of injury. The importance of fractionation, repopulation and dose-rate are considered, followed by "growing-points" in radiotherapy. The latter are mostly based at present on the importance of resistant hypoxic tumour cells, and this leads directly into the next chapter, on hypoxic cell sensitizers (Adams). A lucid account of their development is given by the doyen of this field. The best sensitizer to date remains natural oxygen, and the search is under way for better synthetic sensitizers than "misonidazole". The sensitizer problems of stability, penetration, distribution and toxicity are covered, with an account of trials with humans to date.

Animal tumour models (Kallman and Rockwell) are covered in some detail. Methods for measuring tumour response, both *in vivo* and *in vitro*, are followed by their use in understanding tumour therapy at the cellular level. High LET radiations, *e.g.* neutrons, $\pi$-mesons, are well covered at the physical and cellular level (Hall). The place of such radiations in therapy will become clear in the next decade, as clinical trials are now in progress at many centres. The subsequent chapter on stem cells and non-proliferating cells (Potmesil, LoBue, Goldfeder) would perhaps have been better placed next to the chapter on kinetics. It is a well-referenced chapter, and one of the best attempts yet at comparing normal and malignant tissues at this level. The "ideal" definition of stem cell is still not strictly maintained, as it is applied to committed or transit progenitor cells which have clearly lost their primordial stem-ness.

The role of surgery receives a short overview (Fisher) which covers the ideas of primaries, metastases and cell burdens. The importance of lymph nodes is discussed, followed by the current place of surgery in cancer management.

The section dealing with immunotherapy by Hersh, Mavligit, Gutterman and Richman provides an excellent review of current thinking of the role of this treatment in cancer management. The information is well presented, with many useful summary tables. The relevant studies in animal models are reviewed before dealing with the application of principles to human cancer. Where possible the mode of action of the many agents is discussed, and there are frequent suggestions of areas where further studies are necessary. The authors are to be commended for their attempts to cover the whole range of procedures which can be included under the term immunotherapy, including sections on bacterial products, transfer of immunity with cells and active immunotherapy, as well as immune RNA transfer factor, interferon, thymic hormones and levamisole. The immunotherapy of various disease categories in man (melanoma, lung carcinoma, leukaemia) is also dealt with. This most realistic overview of the field of immunotherapy represents a very real contribution and is to be recommended to all interested in gaining the maximum information in the shortest time.

This is a well-produced volume, and clearly, it will be of use to clinicians and to people moving into these fields.

J. H. Hendry
B. M. Vose